# Ursolic Acid Inhibits Collagen Production and Promotes Collagen Degradation in Skin Dermal Fibroblasts: Potential Antifibrotic Effects

**DOI:** 10.3390/biom15030365

**Published:** 2025-03-03

**Authors:** Tianyuan He, Yaping Xiang, Hehui Quan, Yingchun Liu, Chunfang Guo, Taihao Quan

**Affiliations:** 1 Department of Dermatology, University of Michigan Medical School, Ann Arbor, MI 48109, USA; tyhe@med.umich.edu (T.H.); 600850@csu.edu.cn (Y.X.); yingchun.liu@imau.edu.cn (Y.L.); cfguo@umich.edu (C.G.); 2 Lenox Hill Hospital, 100 E 77th St., New York, NY 10075, USA; hquan@northwell.edu

**Keywords:** ursolic acid, fibrosis, collagen, MMP-1, TGF-β/Smad, MAKP, AP-1

## Abstract

Tissue fibrosis, characterized by excessive collagen accumulation, leads to impaired organ function and is a hallmark of various chronic diseases. Fibroblasts play a central role in collagen production and deposition. This study examines the impact of ursolic acid, a pentacyclic triterpenoid compound present in various fruits and vegetables, on collagen homeostasis in primary human dermal fibroblasts. Ursolic acid (UA) was observed to significantly reduce collagen production while markedly increasing the activity of matrix metalloproteinase-1 (MMP-1), an enzyme responsible for collagen degradation. Mechanistically, ursolic acid was found to inhibit TGF-β/Smad signaling, leading to decreased collagen production, and to activate mitogen-activated protein kinase (MAPK) pathways and activator protein 1 (AP-1), resulting in enhanced MMP-1 production. These in vitro findings were further validated in an in vivo mouse model of fibrosis, where ursolic acid significantly mitigated bleomycin-induced skin fibrosis. These results suggest that UA could be a promising candidate for treating skin fibrosis due to its dual effects on collagen homeostasis: inhibiting collagen production and promoting collagen degradation.

## 1. Introduction

The tissue response to disease and injury results in the deposition of a dense scar through processes collectively known as fibrosis [1]. Fibrosis, marked by the excessive accumulation of collagen, is a common pathological feature of various chronic diseases that affect organs such as the liver, lungs, kidneys, heart, and skin [2,3]. Dysfunctional healing by fibrotic scarring results in structural abnormalities and eventually leads to organ malfunction, leading to lifelong disability, which places a substantial burden on public health [4]. Collectively, fibrosis is implicated in 45% of all deaths in the U.S. [5]. As fibrosis is increasingly recognized as a significant cause of morbidity and mortality in many chronic diseases, effective treatments are emerging in certain conditions. For example, liver fibrosis due to HCV can resolve within 12 months of treatment [6], bone marrow fibrosis can significantly improve after stem cell transplantation [7], and SGLT2 inhibitors have been shown to reduce kidney fibrosis in some patients [8]. These advancements highlight the potential effectiveness of targeted therapies in addressing fibrosis; however, such efforts require a comprehensive understanding of the underlying mechanisms to be effectively developed and implemented. In recent years, researchers have explored natural compounds with potential antifibrotic properties. Ursolic acid (UA) has gained attention for its diverse biological activities, including anti-inflammatory, antioxidant, and potential antifibrotic effects [9,10,11,12]. UA is a pentacyclic triterpenoid compound widely found in various fruits and vegetables [11]. Studies have shown UA antifibrotic activity in liver fibrosis models in rats and mice [12,13,14]. The antifibrotic mechanisms of UA likely involve multiple signaling pathways, such as the inhibition of NFκB, PI3K/Akt, Nrf2/ARE, NOXs/ROS, RhoA/ROCK1, and OX4/NLRP3 inflammasome pathways [12,15,16]. However, the antifibrotic effects of UA are complex and somewhat controversial. While some studies have reported antifibrotic effects of UA [11,15,17,18,19], other research has found limited or no significant antifibrotic action [20,21]. Some studies show reduced collagen deposition with UA treatment [11,17,18,19], suggesting potential antifibrotic effects. However, other research indicates that UA can stimulate the production of collagen [20,21,22,23], particularly types I and III, which are major fibrotic proteins in tissue fibrosis. Despite its potential antifibrotic properties, UA is also used in skincare products aimed at reducing signs of aging and improving skin elasticity [22,24], because of its collagen-boosting properties. As such, its impact on skin collagen and fibrosis remains unclear. These conflicting findings highlight the need for further research to better understand the mechanisms and context-dependent effects of UA on fibrosis and collagen production.

Skin provides an excellent model for studying fibrosis [25]. The human skin dermis primarily consists of a dense, collagen-rich extracellular matrix (ECM), which provides structural and mechanical support [26]. Adult mammalian skin wounds typically heal with fibrotic scars, characterized by the excess deposition of abnormally organized, densely-packed collagen fibrils, a hallmark of fibrosis [4,5]. Dermal fibroblasts, the main cellular component of the dermis, play a crucial role in regulating collagen homeostasis.

In this study, we investigated the effects of UA on collagen homeostasis in the context of fibrosis using human skin primary dermal fibroblasts. We found that UA significantly inhibits collagen production and induces the matrix-degrading protease, matrix metalloproteinase-1 (MMP-1). Mechanistically, UA impairs TGF-β/Smad signaling to inhibit collagen production and activates mitogen-activated protein kinase (MAPK) pathways and activator protein-1 (AP-1) to induce MMP-1. Finally, we demonstrated that UA prevented bleomycin-induced skin fibrosis in a mouse model. These findings suggest that UA is a potential candidate for the treatment of skin fibrosis.

## 2. Materials and Methods

### 2.1. Cell Culture

Skin punch biopsies (full-thickness 4 mm in diameter) were obtained from the sun-protected hip region of healthy adults (mean age 48 ± 5 years, consisting of 2 males and 2 females). The participation of human subjects in this study was approved by the University of Michigan Institutional Review Board (HUM00139214, approved date: 8 April 2022), and all subjects provided written informed consent prior to inclusion. Study exclusion criteria include that all human subjects were HIV negative, and none of the subjects had any systemic or autoimmune diseases, nor were they being treated with steroids or hormonal therapy. Primary human dermal fibroblasts were isolated from human skin biopsies, as described previously [27]. Briefly, the dermis was separated from the epidermis by incubating the tissue in 0.25% trypsin and 0.1% EDTA in phosphate-buffered saline (PBS) for 30 min at 37 °C. The biopsy was then minced into small pieces using scissors and forceps. These tissue fragments were placed in a small dish containing a minimal amount of Dulbecco’s Modified Eagle’s Medium (DMEM) with 4.5 g/L glucose and 2 mM L-glutamine (BioWhittaker, Walkersville, MD, USA), supplemented with 10% (vol/vol) fetal calf serum (Invitrogen, Carlsbad, CA, USA), 100 IU/mL penicillin, and 0.1 mg/mL streptomycin. The limited medium volume allowed tissue pieces to adhere to the plastic surface. Dishes were incubated at 37 °C in an atmosphere of 95% air and 5% CO_2_. After 5–7 days, the tissue was removed, and cells that had migrated from the tissue fragments were observed. Typically, a 4 mm punch biopsy yields 2–3 × 10^5^ fibroblasts. Cells from passages two to nine, in actively growing conditions, were used for experiments. Cells were seeded at 80% confluence the day before each experiment. UA was dissolved in ethanol and cells were exposed to 10 μM UA (Sigma-Aldrich, Burlington, MA, USA) for the indicated times. MEK inhibitor PD98059 (10 μM) and JNK inhibitor SP600125 (10 μM) were purchased from Selleckchem (Munich, Germany) and treated for the indicated times. Control (CTRL) cells were treated with ethanol vehicle.

### 2.2. Cell Viability Assay

Cell viability was assessed using the MTT (3-(4,5-dimethylthiazol-2-yl)-2,5-diphenyltetrazolium bromide) colorimetric assay. Cells were seeded at a density of 5 × 10^3^ cells per well in 96-well culture plates and allowed to adhere overnight. The following day, cells were treated with varying concentrations of UA and incubated for 24 h. After treatment, the medium was replaced with phenol red-free culture medium containing 0.5 mg/mL MTT (Thermo Fisher Scientific, Waltham, MA, USA) and incubated at 37 °C for 4 h to allow the formation of formazan crystals. The resulting purple formazan crystals were solubilized using DMSO, and absorbance was measured at 570 nm using a Victor X4 microplate reader (PerkinElmer, Waltham, MA, USA).

### 2.3. RNA Isolation and Quantitative Real-Time RT-PCR

Total RNA was extracted from cells using the RNeasy Mini Kit (Qiagen, Hilden, Germany) according to the manufacturer’s instructions. Reverse transcription was performed using the TaqMan Reverse Transcription Kit (Applied Biosystems, Foster City, CA, USA). Real-time PCR primers were ordered from RealTimePrimers.com (Col1A1:VHPS-2103,VMPS1277; MMP-1:VHPS-5751; CCN2/CTGF:VHPS-2305; c-Jun:VHPS-4712; c-Fos:VHPS-8854; MMP-3: VMPS-3875; MMP13: VMPS-3866; FN: VHPS-3366, VMPS-2235; ELN:VHPS-2951,VMPS-1913; LAMA5,VHPS-5207,VMPS-3427; Col12A1:VHPS-3366, VMPS-3212; Col6A3,VHPS-2116,VMPS-1290; MMP16, VHPS-5757, and VMPS-3868). Quantitative real-time RT-PCR (qRT-PCR) was conducted using the TaqMan Universal PCR Master Mix (Applied Biosystems, Foster City, CA, USA) on a 7300 Real-Time PCR System (Applied Biosystems, Foster City, CA, USA). To ensure accuracy and reproducibility, PCR reactions were set up using a Biomek 2000 automated liquid handling system (Beckman Coulter, Inc., Brea, CA, USA). All primers and probes were obtained as TaqMan Gene Expression Assays (Applied Biosystems, CA, USA). Multiplex PCR reactions contained primers and probes for the target gene and the housekeeping gene 36B4 (ribosomal protein lateral stalk subunit P0, RPLP0), which served as an internal normalization control for quantitation.

### 2.4. Western Blot Analysis

To prepare whole cell protein extract, cells were lysed in cell extraction buffer (25 mM HEPES [pH 7.7], 0.3 M NaCl, 1.5 mM MgCl_2_, 0.2 mM EDTA, 0.1% Triton X-100, 0.5 mM DTT, 20 mM β-glycerolphosphate, 0.1 mM Na_3_VO_4_, 2 μg/mL leupeptin, and 100 μg/mL PMSF). Cell culture medium was harvested and concentrated using Amicon^®^ Ultra Centrifugal Filter Units (Millipore Sigma, St. Louis, MO, USA). In brief, 1.5 mL of culture medium was loaded into Amicon^®^ Ultra-2 mL centrifugal devices (3 kDa cutoff) and centrifuged at 3000 rpm for 45 min in a fixed-angle rotor. This process resulted in approximately a 15-fold concentration, yielding 100 μL of concentrated medium. Protein concentrations were determined using the Coomassie Plus assay (Pierce Protein Biology, Thermo Fisher Scientific, MA, USA) according to the manufacturer’s instructions. Equal amounts of protein (~50 μg/lane) were loaded onto 6–12% gradient sodium dodecyl sulfate-polyacrylamide (SDS) gels. Proteins were then transferred onto polyvinylidene difluoride membranes and blocked with PBST (0.1% Tween 20 in PBS) containing 5% nonfat milk for one hour at room temperature. Membranes were incubated with primary antibodies for one hour at room temperature. The following primary antibodies were used: type I collagen (SC-293182), MMP-1 (sc-58377), and CCN2/CTGF (sc-101586) (Santa Cruz Biotechnology, Santa Cruz, CA, USA); Smad2/3, p-ERK1/2, c-Jun, and c-Fos (Cell Signaling Technology, MA, USA); and β-actin (Sigma, St. Louis, MO, USA). After washing three times with PBST, the membranes were incubated with appropriate secondary antibodies (anti-rabbit and anti-goat monoclonal secondary antibodies, Santa Cruz Biotechnology, CA, USA) for one hour at room temperature. Following three additional washes with PBST, the membranes were developed using the Vistra ECF Western blotting system (GE Healthcare, Piscataway, NJ, USA) according to the manufacturer’s instructions. The membranes were then scanned using a STORM PhosphorImager (Molecular Dynamics, Sunnyvale, CA, USA). Band intensities were measured using ImageQuant software (version 2.1.0/1.53h) and normalized to β-actin as a loading control. Original western blots can be found at Supplementary Materials.

### 2.5. Transient Transfection and Luciferase Assays

Human primary skin fibroblasts were transiently transfected by electroporation using the Amaxa system (Lonza, Basel, Switzerland). Transfections were carried as previously described [28,29]. Transient transfection of Emerald Green Fluorescent Protein (EmGFP, ThermoFisher, Waltham, MA, USA) demonstrated transfection efficiency of up to 80% in human primary skin fibroblasts using this method [23,28]. For luciferase assays, 1 × 10^6^ primary human dermal fibroblasts were electroporated with an AP-1 reporter construct (pAP1-TA-Luc, BD Biosciences Clontech, Palo Alto, CA, USA) or SBE 4x reporter construct [30]. Cells were co-transfected with a β-galactosidase expression vector to provide an internal control for transfection efficiency. Aliquots containing identical β-galactosidase activity were used for each luciferase assay. Luciferase activity was measured using an enhanced luciferase assay kit (BD Biosciences, San Diego, CA, USA) following the manufacturer’s protocol.

### 2.6. Mouse Model of Skin Fibrosis

Eight-week-old female C57BL/6J (strain# 000664) mice were purchased from the Jackson Laboratory (Bar Harbor, ME, USA). All animal experiments were reviewed and approved by the review board for animal experiments of University Michigan (The Unit for Laboratory Animal Medicine) (Approved code: PRO00009530, Approved date: 6 May 2020). Mice were intradermally injected with bleomycin (100 μL, 1 mg/mL, Sigma-Aldrich, Burlington, MA, USA) and ursolic acid (UA) (100 μL, 20 μM) topically for three weeks. Briefly, bleomycin was intradermally injected into the shaved back skin every other day. UA was topically applied on alternate days between bleomycin treatments. At the end of the study, back skin was collected, and H&E stained to quantify dermal thickness. Mouse skin (50 μm thickness) collagen fibers were visualized using second harmonic generation (SHG) microscopy (Leica, Wetzlar, Germany), which generates signals exclusively from non-centrosymmetric structures such as fibrillar collagen. SHG images were acquired using a Leica SP8 Confocal Microscope (Leica, Wetzlar, Germany, laser excitation wavelength: 800 nm, recorded emission wavelength: 400 nm) with 2-Photon at the University of Michigan Microscopy and Image Analysis Laboratory. To account for spatial heterogeneity, we analyzed multiple fields of view per sample. Raw SHG images were processed using ImageJ software (NIH, FIJI-Win64, version 2.1.0/1.53h), with the background signal subtracted using measurements from adjacent regions devoid of visible collagen, and pixel intensity values were calibrated to quantify collagen density. Collagen degradation was determined by collagen hybridizing peptide (CHP) (3Helix Inc. Salt Lake City, UT, USA), which is a novel and unique peptide that specifically binds denatured and unfolded collagen chains. OCT-embedded skin sections were stained with CHP (conjugated to a fluorescent dye, 5-FAM) and imaged using fluorescence microscopy.

### 2.7. Charts and Statistics

The data were organized in Microsoft Excel 365, and then transferred into GraphPad Prism (v.8) for statistical analysis and graph generation. All data are represented as mean ± SEM. Statistical analysis was performed using GraphPad Prism (v.8) with unpaired two-sided Student’s *t*-tests, one-way analysis of variance (ANOVA) with Tukey’s method for multiple comparisons, or Kruskal–Wallis test with Dunn’s multiple comparisons test. Statistical significance was defined as *p* < 0.05. All experiments were repeated a minimum of three times unless otherwise stated.

## 3. Results

### 3.1. UA Suppresses Type I Collagen Expression and Stimulates MMP-1 Expression

**Figure 1:**
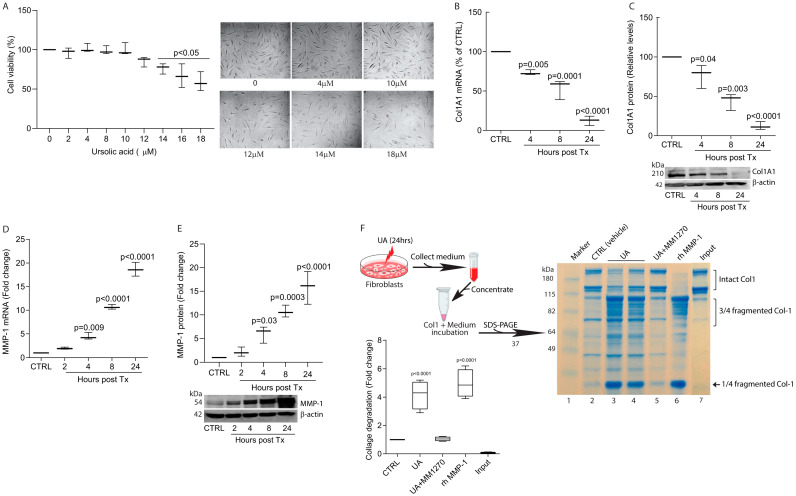
UA suppresses type I collagen expression and stimulates MMP-1 expression in primary dermal fibroblasts. (**A**) Cell viability assay. Cell viability was assessed using the MTT colorimetric assay. Phase contrast images were shown. (**B**–**E**) Cells were treated with UA (10 μM) for the indicated time periods. (**B**) Col1A1 mRNA, (**C**) Col1A1 protein, (**D**) MMP-1 mRNA, and (**E**) MMP-1 protein levels. mRNA levels were quantified by real-time RT-PCR and normalized to 36B4 mRNA, a ribosomal protein used as an internal control. Protein levels were determined by Western blot analysis and normalized to β-actin as a loading control. Data are expressed as mean ± SEM (N = 3). *p*-values are compared to the control (CTRL) and are displayed in the respective graphs. (**F**) Collagen fragmentation by UA: Conditioned media were collected, concentrated, and incubated with rat tail type I collagen. The reaction products were resolved by 10% SDS-PAGE. Lane 1: marker, Lane 2: CTRL (vehicle), Lane 3 and 4: UA, Lane 5: UA and MMP1270 (MMP inhibitor), Lane 6: rh-MMP-1, Lane 7: Type I collagen input. Intact and fragmented collagens were visualized by SimplyBlue staining and quantified ImageJ software (NIH, FIJI-Win64, version 2.1.0/1.53h). Data are expressed as mean ± SEM (N = 4). *p*-values are compared to the control (CTRL). Original western blots can be found at Supplementary Materials.

We initially explored the impact of UA on type I collagen (Col1A1), a key structural protein in the skin dermis, using primary human skin dermal fibroblasts. We first assessed the cytotoxicity of UA across a range of concentrations. Cell viability decreased at UA concentrations above 10 μM (Figure 1A). Based on these results, we selected 10 μM UA for subsequent experiments. Time-course analysis demonstrated that UA significantly reduced Col1A1 mRNA (Figure 1B) and protein levels (Figure 1C), the major structural protein in skin, in a time-dependent manner. After 24 h of treatment, UA decreased Col1A1 mRNA (Figure 1B) and protein (Figure 1C) levels by approximately 88%. Next, we investigated the effect of UA on matrix metalloproteinase-1 (MMP-1), the major collagen-degrading protease in skin [31]. UA significantly induced MMP-1, in a time-dependent manner (Figure 1D,E). At 24 h post-treatment, UA induced MMP-1 nearly 20-fold in both MMP-1 mRNA (Figure 1D) and protein (Figure 1E). Next, we collected and concentrated cell culture medium to assess the impact of UA-induced MMP-1 secretion on collagen degradation. Medium from UA-treated cells generated one-quarter and three-quarter length collagen fragments (Figure 1F, lanes 3 and 4), which are characteristic of MMP-1 activity [32,33]. This was confirmed by comparing the fragments to those produced by treatment with recombinant human MMP-1 (Figure 1F, lane 6 and bar graph). UA-mediated collagen fibril fragmentation was completely blocked by the MMP inhibitor MMI270 (Figure 1F, lane 5 and bar graph), demonstrating the specificity of the collagen fragmentation process. These data suggest that UA regulates dermal fibroblast collagen homeostasis by both inhibiting collagen expression and promoting its degradation.

### 3.2. UA Inhibits Type I Collagen Expression by Impairing TGF-β/Smad Signaling

**Figure 2:**
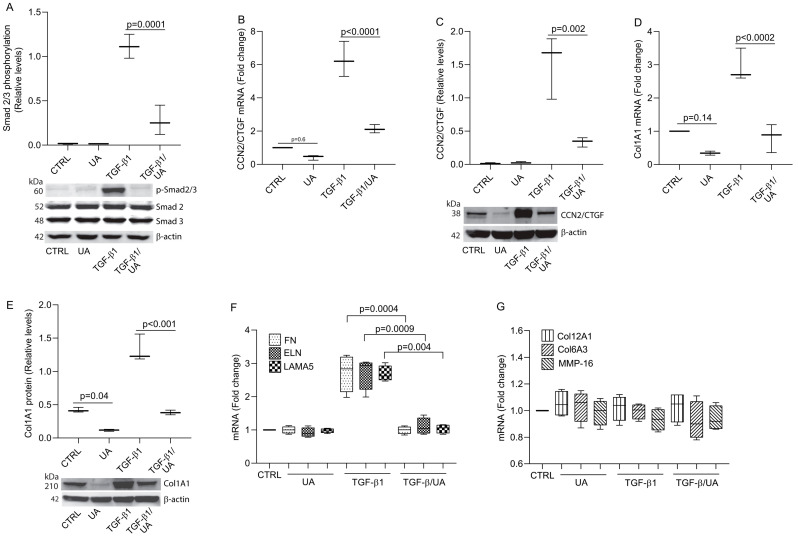
UA inhibits type I collagen expression by impairing TGF-β/Smad signaling in primary dermal fibroblasts. (**A**) UA inhibits TGF-β1-induced Smad2/3 phosphorylation. Cells were treated with UA (10 μM) for 4 h, followed by TGF-β1 (5 ng/mL) for one hour. Smad2/3 phosphorylation was assessed by Western blot. (**B**–**E**) Cells were treated with UA (10 μM) and TGF-β1 (5 ng/mL) for 24 h. UA inhibits TGF-β1-induced expression of (**B**) CCN2/CTGF mRNA, (**C**) CCN2/CTGF protein, (**D**) Col1A1 mRNA, (**E**) Col1A1 protein, and (**F**) FN, ELN, and LAMA5 mRNA. (**G**) Non-TGF-β target genes remain unaffected by UA treatment. mRNA levels were quantified by real-time RT-PCR and normalized to 36B4 mRNA (internal control). Protein levels were determined by Western blot analysis and normalized to β-actin (loading control). Data are expressed as mean ± SEM (N = 3). *p*-values are indicated in the respective graphs. Original western blots can be found at Supplementary Materials.

Next, we investigate potential mechanisms by which UA inhibits Col1A1 expression. Among the multitude of signaling pathways involved in collagen regulation, TGF-β/Smad signaling is considered a principal pathway for controlling collagen expression [34]. The canonical TGF-β pathway involves the phosphorylation of Smad2/3, which then forms a complex with Smad4 and translocates to the nucleus to regulate target gene transcription [35]. Our results demonstrate that UA treatment significantly reduced the phosphorylation levels of Smad2/3, while total Smad2/3 protein levels remained unchanged (Figure 2A). We investigated the effects of UA on CCN2/CTGF expression, a critical downstream mediator of the TGF-β/Smad pathway that plays an essential role in regulating collagen synthesis [29]. We found that while TGF-β treatment robustly induced CCN2/CTGF expression, co-treatment with UA significantly attenuated this response at both the mRNA (Figure 2B) and protein levels (Figure 2C). This inhibitory effect extended to Col1A1, where UA treatment markedly suppressed TGF-β-induced Col1A1 mRNA (Figure 2D) and protein expression (Figure 2E). Western blot analysis revealed that UA reduced CCN2/CTGF protein levels by approximately 80% compared to TGF-β treatment alone, with a corresponding 75% reduction in Col1A1 protein expression. Since TGF-β signaling regulates the expression of numerous ECM genes, we investigated the effect of UA on other TGF-β-regulated genes, including fibronectin (FN), elastin (ELN), and laminin subunit alpha-5 (LAMA5), which is a major one of the major membrane proteins in skin [36,37]. UA significantly inhibited TGF-β-induced FN, ELN, and LAMA5 mRNA expression, without affecting basal expression (Figure 2F). Furthermore, non-TGF-β target genes, such as Col12A1, Col6A3, and MMP16, remain unaffected by UA treatment (Figure 2G). These findings indicate the specificity of UA effects on a subset of TGF-β-regulated genes. Collectively, these data suggest that UA impairs TGF-β/Smad signaling, thereby inhibiting a broad range of TGF-β-regulated ECM genes, including type I collagen, the major structural protein in skin.

### 3.3. UA Induces MMP-1 by Activation of MAPK Pathways

**Figure 3:**
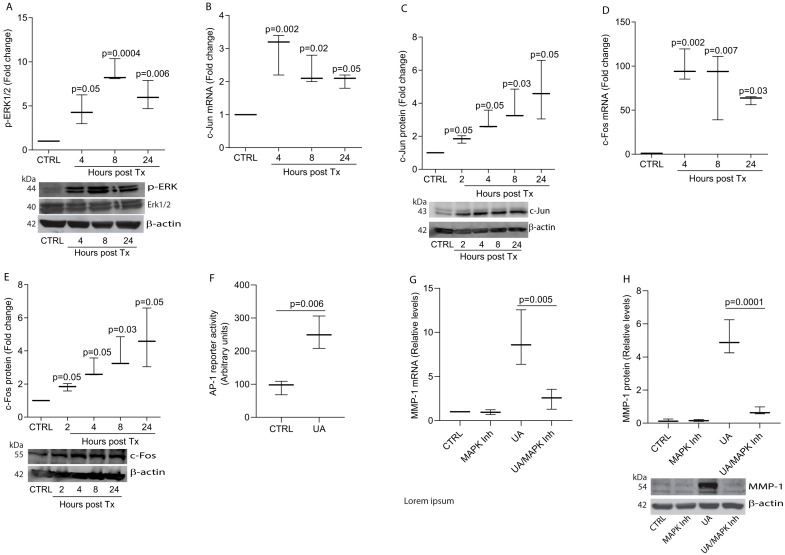
UA induces MMP-1 by activation of MAPK pathways in primary dermal fibroblasts. Cells were treated with UA (10 μM) for the indicated time periods. (**A**) ERK1/2 activation, (**B**) c-Jun mRNA, (**C**) c-Jun protein, (**D**) c-Fos mRNA, and (**E**) c-Fos protein levels. (**F**) UA increases AP-1 reporter activity. Cells were transiently transfected with AP-1 reporter construct and AP-1 activity was determined by luciferase assay. (**G**,**H**) UA-induced MMP-1 expression was blocked by MAPK inhibitors. Cells were treated with MAPK (PD98059 and SP600125 for 2 h and then treated with UA (10 μM) for 24 h. (**G**) MMP-1 mRNA and (**H**) MMP-1 protein. mRNA levels were quantified by real-time RT-PCR and normalized to 36B4 mRNA, a ribosomal protein used as an internal control. Protein levels were determined by Western blot analysis and normalized to β-actin as a loading control. Data are expressed as mean ± SEM (N = 3). *p*-values are shown in the respective graphs. Original western blots can be found at Supplementary Materials.

Since MMP-1 expression is predominantly regulated through MAPK pathways [38], we investigated whether UA upregulation of MMP-1 involves the activation of MAPK pathways. UA treatment activated ERK1/2 (Figure 3A) and induced the expression of AP-1 components c-Jun (Figure 3B, mRNA; Figure 3C, protein) and c-Fos (Figure 3D, mRNA; Figure 3E, protein). The AP-1 reporter assay further revealed that UA significantly enhanced AP-1 transcriptional activity (Figure 3F). To determine whether MAPK signaling was necessary for UA-induced MMP-1 expression, we treated cells with MAPK inhibitors. MAPK inhibition almost completely abolished UA-induced MMP-1 expression at both mRNA (Figure 3G) and protein (Figure 3H) levels. These results demonstrate that UA upregulates MMP-1 expression through the activation of MAPK signaling pathways.

### 3.4. UA Inhibits Skin Thickness in Bleomycin-Induced Mouse Model of Fibrosis

**Figure 4:**
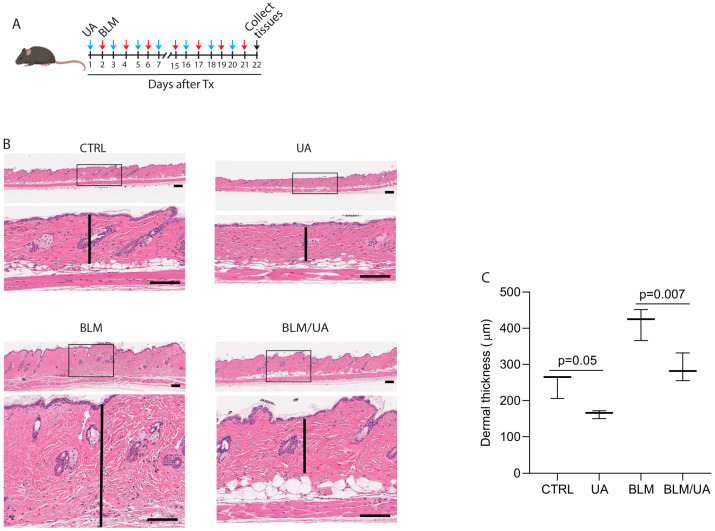
UA inhibits skin thickness in bleomycin-induced mouse model of fibrosis. (**A**) Experimental design: C57BL/6J mice were intradermally treated with bleomycin (100 μL, 1 mg/mL) subcutaneously every other day and UA (100 μL, 20 μM) topically on alternate days for three weeks. (**B**) UA treatment reduces bleomycin-induced dermal thickening. Representative H&E staining of back skin. Control mice were treated with the solvent ethanol. Scale bars = 100 μm. N = 5 in each group. (**C**) Dermal thickness was quantified using ImageScope (version 12.4.6, Leica, Deer Park, IL, USA). N = 5 in each group. Data are expressed as mean ± SEM (N = 5 in each group). *p*-values are shown in the respective graphs.

Given the ability of UA to inhibit collagen expression and promote collagen degradation, we explored its antifibrotic potential using a well-established bleomycin-induced mouse model of skin fibrosis [39]. Mice received alternating treatments over three weeks: intradermal bleomycin (100 μL, 1 mg/mL) injections every other day, with topical UA (100 μL, 20 μM) applications on intervening days (Figure 4A). As expected, bleomycin injection significantly increased dermal thickness, indicating fibrosis (Figure 4B bottom left and Figure 4C bar graph). Notably, topical UA treatment markedly reduced this bleomycin-induced skin thickening (Figure 4B bottom right panel and Figure 4C bar graph). Additionally, topical UA alone decreased skin dermal thickness in untreated control mice (Figure 4B top right panel and Figure 4C bar graph). These data suggest that UA inhibits skin thickness in the bleomycin-induced mouse model of fibrosis.

### 3.5. UA Inhibits Collagen Production by Impairing TGF-β/Smad Signaling in Bleomycin-Induced Mouse Model of Fibrosis

**Figure 5:**
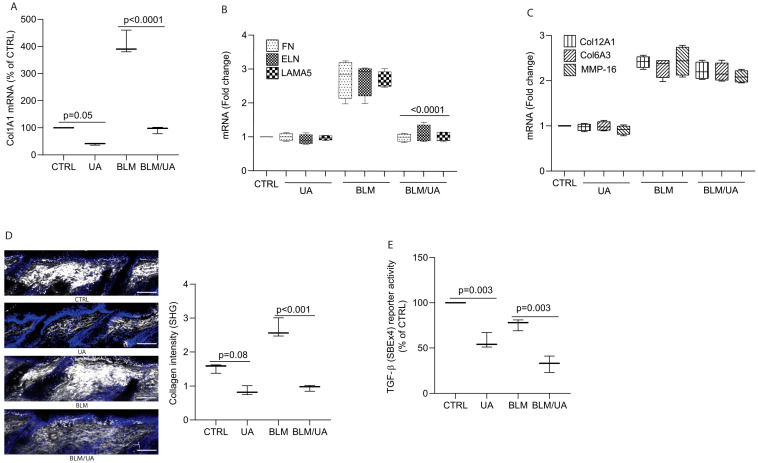
UA inhibits collagen production by impairing TGF-β/Smad signaling in bleomycin-induced mouse model of fibrosis. C57BL/6J mice were intradermally treated with bleomycin (100 μL, 1 mg/mL) subcutaneously every other day and UA (100 μL, 20 μM) topically on alternate days for three weeks. (**A**) UA inhibits Col1A1 mRNA expression. (**B**) UA inhibits FN, ELN, and LAM mRNA expression. *p*-values are compared to the bleomycin (BLM). (**C**) Non-TGF-β target genes remain unaffected by UA treatment. mRNA levels were quantified by real-time RT-PCR and normalized to 36B4 mRNA, a ribosomal protein used as an internal control. Data are expressed as mean ± SEM (N = 5 in each group). *p*-values are shown in the respective graphs. (**D**) UA treatment decreases dermal collagen intensity. Second harmonic generation (SHG) microscopy images. White signals represent SHG from collagen fibers. SHG signals were quantified ImageJ software (NIH, FIJI-Win64). Data are expressed as mean ± SEM (N = 5 in each group). *p*-values are shown in the respective graphs. Scale bars = 100 μm. (**E**) UA inhibits TGF-β/Smad signaling. SBE 4x reporter activity was determined by luciferase assay. Data are expressed as mean ± SEM (N = 5 in each group). *p*-values are shown in the respective graphs.

The above data raised the question of whether UA could reduce bleomycin-induced collagen production and accumulation in the dermis of mouse skin. To explore this, we first determined Col1A1 mRNA expression. We found that while bleomycin treatment robustly induced Col1A1 expression, co-treatment with UA significantly attenuated this response (Figure 5A). In addition to Col1A1, UA significantly inhibited other TGF-β target genes, FN, ELN, and LAMA5 mRNA expression, without affecting basal expression (Figure 5B). Furthermore, non-TGF-β target genes such as Col12A1, Col6A3, and MMP-16, remain unaffected by UA treatment (Figure 5C), suggesting the specificity of UA effects on a subset of TGF-β-regulated genes. Next, we assessed dermal collagen fiber density using two-photon microscopy. While bleomycin significantly increased collagen fiber density (Figure 5D, third panel from the top and bar graph), UA treatment markedly reduced bleomycin-induced dermal collagen accumulation (Figure 5D, bottom panel and bar graph). Additionally, topical UA alone decreased skin dermal collagen fiber density in untreated control mice (Figure 5D, second panel from top and bar graph). We extended our in vitro findings that UA could inhibit bleomycin-induced collagen production by the impairment of TGF-β/Smad signaling (Figure 2). To test this possibility, we conducted a well-established SBE 4x (tandem repeats of Smad Biding Element) reporter assay, which utilizes tandem repeats of the Smad Binding Element (SBE) as a highly sensitive tool to assess TGF-β-induced Smad transcriptional activity [30]. Our results confirmed that UA treatment significantly reduced SBE 4x reporter activity in either UA alone or bleomycin-treated skin (Figure 5E). These findings suggest that UA may inhibit bleomycin-induced collagen production and accumulation by suppressing TGF-β/Smad signaling.

### 3.6. UA Promotes Collagen Degradation by Activating AP-1 in Bleomycin-Induced Mouse Model of Fibrosis

**Figure 6:**
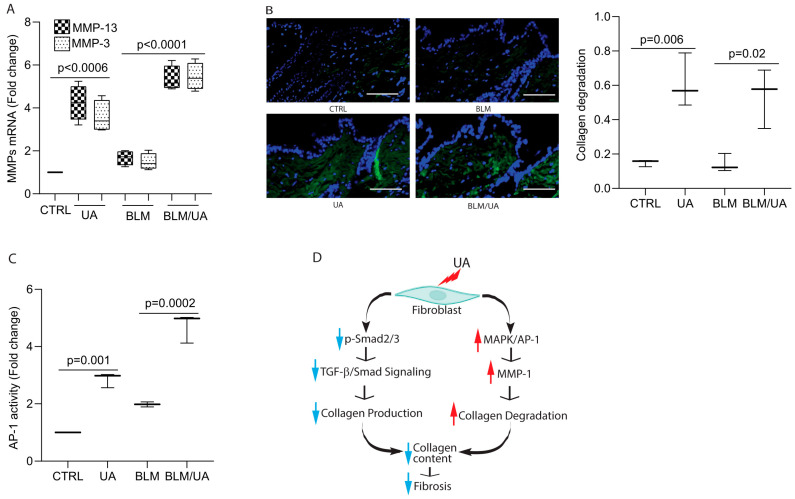
UA promotes collagen degradation by activating AP-1 in bleomycin-induced mouse model of fibrosis. C57BL/6J mice were intradermally treated with bleomycin (100 μL, 1 mg/mL) subcutaneously every other day and UA (100 μL, 20 μM) topically on alternate days for three weeks. (**A**) UA upregulates mouse collagenases, MMP-13 and MMP-3, mRNA expression. mRNA levels were quantified by real-time RT-PCR and normalized to 36B4 mRNA, a ribosomal protein used as an internal control. Data are expressed as mean ± SEM (N = 5 in each group). *p*-values are shown in the respective graphs. (**B**) UA treatment enhances dermal collagen degradation (green signals). Collagen degradation was determined by collagen hybridizing peptide (CHP), which specifically binds denatured and unfolded collagen chains. Data are expressed as mean ± SEM (N = 5 in each group). *p*-values are shown in the respective graphs. Scale bars = 100 μm. (**C**) UA induces AP-1 reporter activity. AP-1 reporter activity was determined by luciferase assay. Data are expressed as mean ± SEM (N = 5 in each group). *p*-values are shown in the respective graphs. (**D**) Proposed mechanism: Schematic representation of UA effects on skin fibrosis. UA exerts a dual action on collagen homeostasis: (1) Inhibition of collagen production: UA impairs TGF-β/Smad signaling pathway, a critical regulator of collagen synthesis, thereby reducing collagen production. (2) Promotion of collagen degradation: UA activates the MAPK/AP-1 pathway, leading to the upregulation of MMP-1, a key enzyme involved in collagen breakdown. This combined action suggests that UA could be an effective antifibrotic agent in tissue fibrosis.

Next, we evaluated collagen degradation in bleomycin-injected mouse skin following UA treatment. We first determined the mRNA expression of two major mouse collagenases, MMP-13 and MMP-3, as mice lack MMP-1 expression. We confirmed that the mRNA levels of both MMP-13 and MMP-3 were significantly upregulated in the skin of mice treated with either UA alone or bleomycin (Figure 6A). To assess collagen degradation, we used staining with a collagen hybridizing peptide (CHP) that binds specifically to denatured and unfolded collagen chains. We observed significant collagen degradation in the skin of both UA-treated mice (Figure 6, lower left panel and bar graph) and bleomycin-treated mice (Figure 6, lower right panel and bar graph). To explore the mechanism underlying UA-mediated collagen degradation, we conducted an AP-1 reporter assay, as the AP-1 complex is a key regulator of multiple MMPs [38]. We found that UA treatment enhanced AP-1 reporter activity in both untreated control and bleomycin-injected skin (Figure 6C, bottom panels and bar graph). These findings suggest that UA may induce MMP expression via AP-1 activation, facilitating collagen degradation in mouse skin.

## 4. Discussion

In this study, we investigated the effects of UA on collagen homeostasis in the context of skin fibrosis. Our findings demonstrate that UA exhibits potent antifibrotic properties through dual mechanisms: the inhibition of collagen production and stimulation of collagen degradation. These effects were observed both in vitro using human dermal fibroblasts (Figure 1, Figure 2 and Figure 3) and in vivo using a bleomycin-induced mouse model of skin fibrosis (Figure 4, Figure 5 and Figure 6). The role of UA in collagen regulation has been a subject of controversy in the literature. While some studies have reported antifibrotic effects [11,15,17,18,19], others have suggested that UA can stimulate collagen production, particularly in the context of skincare products aimed at reducing signs of aging [20,21,22,23].

Our results illustrate the antifibrotic potential of UA, at least in the context of skin fibrosis. We demonstrate that UA significantly suppresses type I collagen expression at both mRNA and protein levels in dermal fibroblasts. This finding is particularly relevant given that type I collagen is the major structural component of the skin dermis and a key ECM protein in fibrosis. Mechanistically, we show that UA impairs the TGF-β/Smad signaling pathway, which is widely recognized as a principal regulator of collagen expression [34]. UA almost completely suppressed TGF-β-induced Smad2/3 phosphorylation and blocked the expression of CCN2/CTGF, a crucial downstream mediator of TGF-β signaling in fibrosis [29,40,41]. This mechanism of action is significant, as targeting the TGF-β/Smad pathway has been a focus of antifibrotic therapy development [42]. Importantly, our study reveals that UA not only inhibits collagen production but also promotes its degradation through the upregulation of MMP-1. This dual action on collagen homeostasis is particularly promising for antifibrotic therapies. The induction of MMP-1 by UA was substantial, with a nearly 20-fold increase in both mRNA and protein levels. Functional assays confirmed that this increase in MMP-1 results in enhanced collagen degradation. The ability of UA to stimulate collagen breakdown while simultaneously inhibiting its production suggests a convincing mechanism for reversing established fibrosis, a major challenge in treating fibrotic diseases. We further elucidate that UA induces MMP-1 expression through the activation of MAPK pathways, leading to increased AP-1 activity. This finding adds to our understanding of UA molecular mechanisms and highlights its multifaceted effects on cellular signaling pathways relevant to fibrosis.

The in vivo efficacy of UA in preventing bleomycin-induced skin fibrosis in mice provides compelling evidence for its therapeutic potential. The topical application of UA not only reduced dermal thickening and collagen fiber density in the fibrosis model but also decreased dermal thickness in normal skin. This observation suggests that UA might have applications beyond treating established fibrosis, potentially in preventing excessive scarring or maintaining normal skin homeostasis.

Our findings contribute to resolving the conflicting reports on the effects of UA on collagen. The antifibrotic effects of UA observed in our study appear to be incongruent with its previously reported collagen-enhancing properties in skincare applications. This apparent contradiction might be explained by context-dependent effects of UA, possibly influenced by factors such as concentration, the route of administration, or the physiological state of the target tissue. Further research is needed to fully elucidate these context-dependent effects.

UA has similarity to the related triterpene compounds, such as oleanolic acid (OA) and betulinic acid (BA). As the antifibrotic effects of OA and BA have been previously reported [43,44], we compared the antifibrotic properties of UA with those of other triterpene compounds, including OA and BA. We found that while OA and BA also inhibit type I collagen expression in human dermal fibroblasts, they do not affect MMP-1 expression (see Supplemental Figure S1). Moreover, their inhibitory effects on type I collagen are significantly less potent than those of UA. These findings highlight the unique properties of UA, including its dual effects on collagen homeostasis: simultaneously inhibiting collagen production and promoting collagen degradation. Several limitations and areas for future research should be noted. First, the long-term effects and safety profile of UA treatment need to be evaluated. For example, mechanisms that encourage apoptosis in skin cells or trigger inflammatory pathways could be detrimental, especially upon high doses or long-term application. A balanced evaluation of UA benefits versus its risks is essential, particularly considering side effects associated with prolonged topical use, such as irritation, atrophy, and potential systemic impacts. Second, the optimal dosing and delivery methods for UA in treating skin fibrosis should be determined. Systematic studies focusing on dosage, context, and long-term effects remain essential for clarifying the exact nature of UA’s influence on skin. For example, investigating whether a lower dose and duration of UA could selectively target fibrotic tissue without affecting normal skin. Third, investigating the effects of UA on other cell types involved in fibrosis, such as immune cells and endothelial cells, would provide a more comprehensive understanding of its antifibrotic mechanism. Lastly, while our findings demonstrate that UA exerts its antifibrotic effects primarily through the TGF-β/Smad and MAPK signaling pathways in skin fibrosis, they do not rule out the potential involvement of other mechanisms. For instance, the pleiotropic nature of the MAPK pathway activated by UA raises questions regarding its specificity. UA reduces skin thickness in both normal and bleomycin-induced fibrotic skin. Investigating UA’s effects on ECM components and signaling pathways in normal versus fibrotic skin could provide valuable insights into the underlying mechanisms. Comprehensive studies are needed to fully elucidate its mechanisms of action and potential off-target effects. In conclusion, our study demonstrates that UA exerts potent antifibrotic effects through dual mechanisms of collagen regulation: the inhibition of production via TGF-β/Smad signaling impairment and stimulation of degradation via MMP-1 induction via the activation of MAPK pathway. These findings position UA as a promising candidate for the treatment of skin fibrosis and potentially other fibrotic disorders. Further research into the clinical applications of UA could open new avenues for addressing the significant unmet medical need in fibrosis treatment.

## 5. Conclusions

Our findings highlight the antifibrotic effects of ursolic acid through its dual regulation of collagen homeostasis, both by inhibiting collagen production and promoting collagen degradation. Mechanistically, ursolic acid suppresses TGF-β/Smad signaling, reducing collagen synthesis, while simultaneously activating the MAPK pathway and AP-1, leading to increased MMP-1 production and enhanced collagen degradation. These in vitro results were further validated in an in vivo mouse model of fibrosis, where ursolic acid significantly alleviated bleomycin-induced skin fibrosis. Collectively, these findings suggest that ursolic acid holds promise as a potential therapeutic agent for skin fibrosis by effectively modulating collagen balance.

## Data Availability

Data are contained within the article.

## Supplementary Materials

The following supporting information can be downloaded at: https://www.mdpi.com/article/10.3390/biom15030365/s1, Figure S1: Oleanolic acid (OA) and betulinic acid (BA) inhibit type I collagen expression in human dermal fibroblasts without affecting MMP-1 expression. Cells were treated with UA, OA, or BA (10 μM) for 24 h. Col1A1 and MMP-1 mRNA levels were quantified by real-time RT-PCR and normalized to 36B4 mRNA, a ribosomal protein used as an internal control. Data are expressed as mean *±* SEM (N *=* 3). *p*-values are compared to the control (CTRL); File S1: Original western blots.
