# Supplementary material for: Ursolic Acid Inhibits Collagen Production and Promotes Collagen Degradation in Skin Dermal Fibroblasts: Potential Antifibrotic Effects
Source: Biomolecules. 2025 Mar 3;15(3):365. doi: 10.3390/biom15030365 (PMC11939892; doi:10.3390/biom15030365)
Supplement: Supplementary file 1 [file biomolecules-15-00365-s001.zip › biomolecules-3407842-Figure S1.pdf]

biomolecules

MDPI

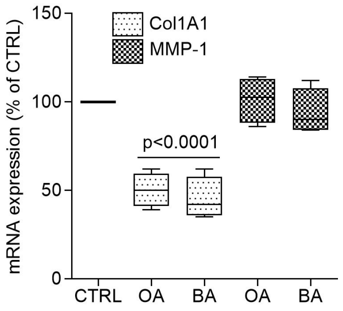

Supplemental Figure S1. Oleanolic acid (OA) and betulinic acid (BA) inhibit type I collagen expression in human dermal fibroblasts without affecting MMP-1 expression. Cells were treated with UA, OA, or BA (10μM) for 24 hours. Col1A1 and MMP-1 mRNA levels were quantified by real-time RT-PCR and normalized to 36B4 mRNA, a ribosomal protein used as an internal control. Data are expressed as mean±SEM (N=3). p-values are compared to the control (CTRL).
