# Supplementary figures and images for: Ursolic Acid Inhibits Collagen Production and Promotes Collagen Degradation in Skin Dermal Fibroblasts: Potential Antifibrotic Effects
Source: Biomolecules. 2025 Mar 3;15(3):365. doi: 10.3390/biom15030365 (PMC11939892; doi:10.3390/biom15030365)

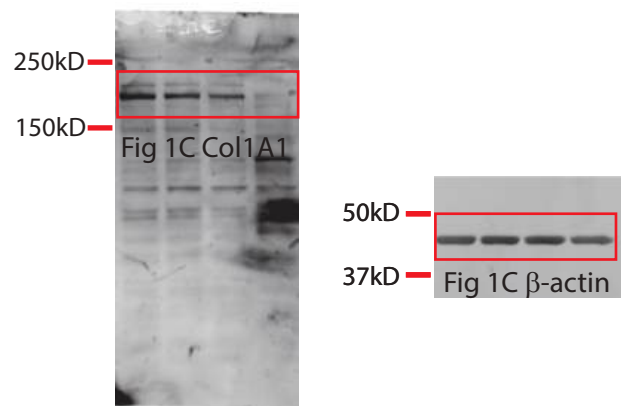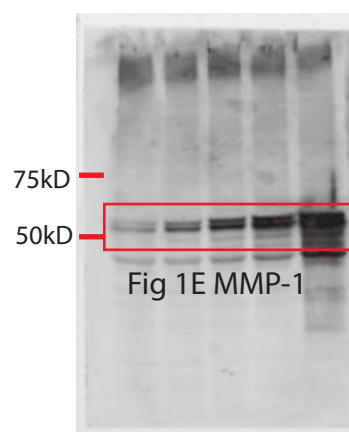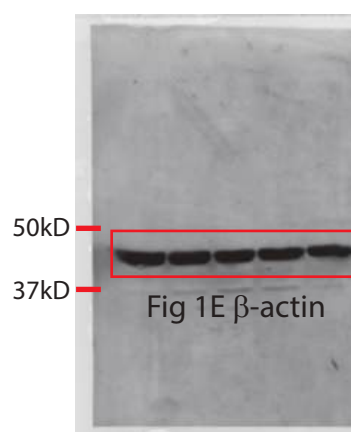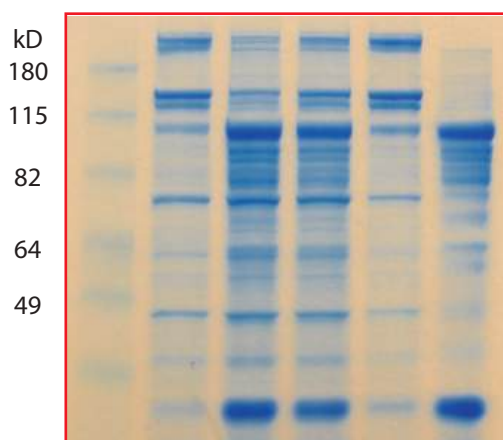

Fig 1F Lanes 1-6

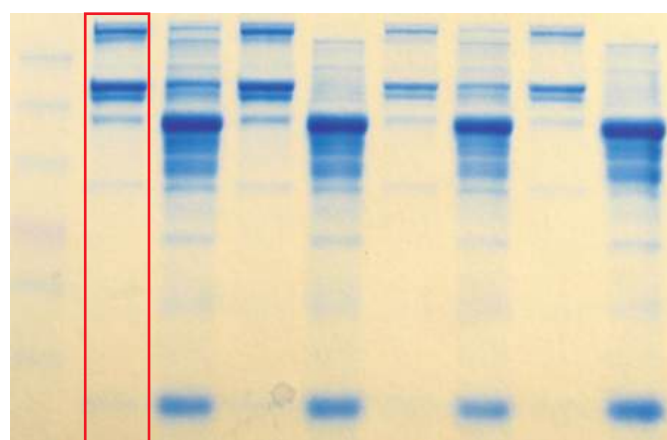

Fig 1F Lane 7

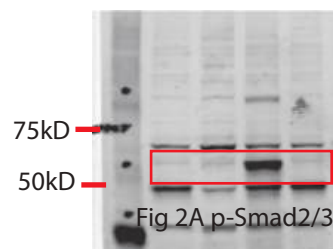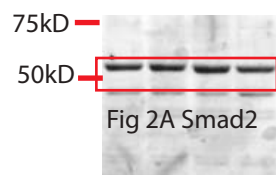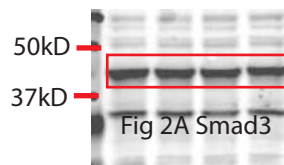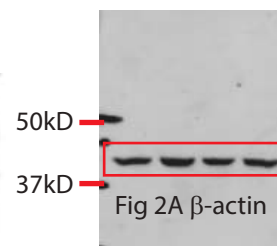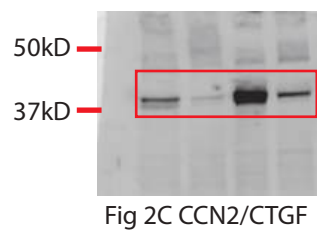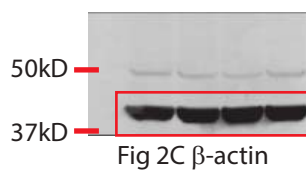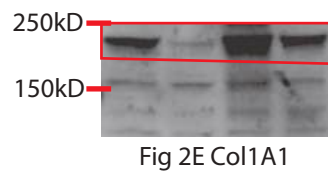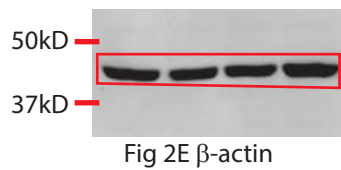

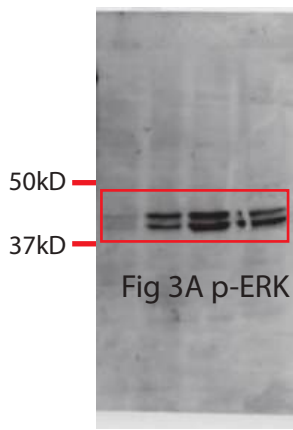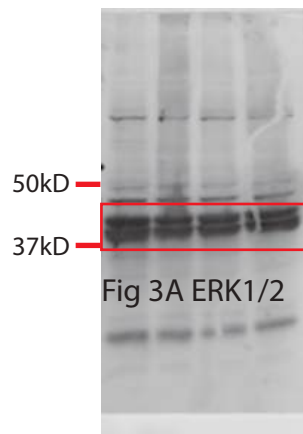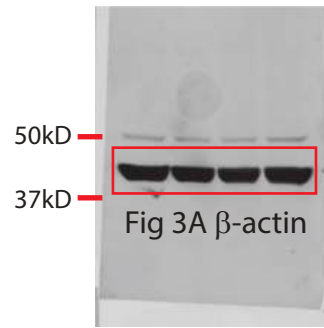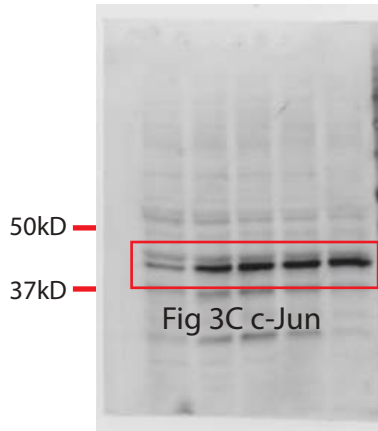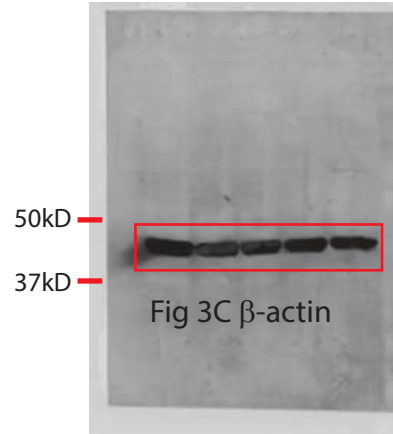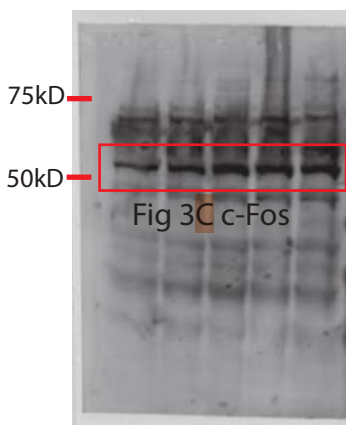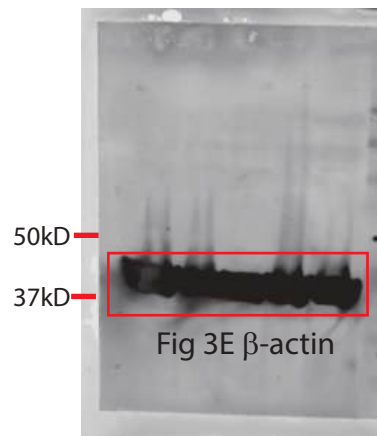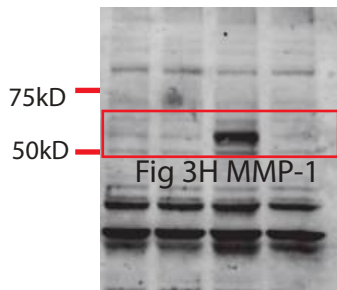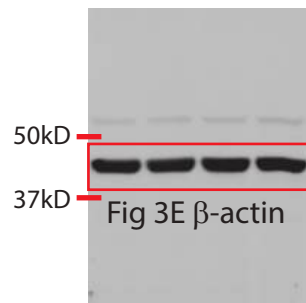

Supplement: Supplementary file 1 [file biomolecules-15-00365-s001.zip › biomolecules-3407842-File S1/biomolecules-3407842UA Ms original gel files.pdf]
